# Transport Properties of Protic Ionic Liquids Based on Triazolium and Imidazolium: Development of an Air-Free Conductivity Setup

**DOI:** 10.3390/molecules28135147

**Published:** 2023-06-30

**Authors:** Eduardo Maurina Morais, Alexander Idström, Lars Evenäs, Anna Martinelli

**Affiliations:** Department of Chemistry and Chemical Engineering, Chalmers University of Technology, 412 96 Gothenburg, Sweden

**Keywords:** protic ionic liquids, imidazolium, triazolium, self-diffusion, ionic conductivity, fragility

## Abstract

The dynamical properties of four protic ionic liquids, based on the ethyltriazolium ([C${}_{2}$HTr${}_{124}$]) and the ethylimidazolium ([C${}_{2}$HIm]) cation, were investigated. The associated anions were the triflate ([TfO]) and the bistriflimide ([TFSI]). Ionic conductivity values and self-diffusion coefficients were measured and discussed, extending the discussion to the concept of fragility. Furthermore, in order to allow the measurement of the ionic conductivity of very small volumes (<0.5 mL) of ionic liquid under an inert and dry atmosphere, a new setup was developed. It was found that the cation nature strongly affected the transport properties, the [C${}_{2}$HTr${}_{124}$] cation resulting in slower dynamics than the [C${}_{2}$HIm] one. This was concluded from both conductivity and diffusivity measurements while for both properties, the anion had a lesser effect. By fitting the conductivity data with the Vogel–Fulcher–Tammann (VFT) equation, we could also estimate the fragility of these ionic liquids, which all fell in the range of very fragile glass-forming materials. Finally, the slower dynamics observed in the triazolium-based ionic liquids can be rationalized by the stronger interactions that this cation establishes with both anions, as deduced from the frequency analysis of relevant Raman signatures and density functional theory (DFT) calculations.

## 1. Introduction

Ionic liquids are a fascinating class of materials that offer a set of useful properties. These include a low volatility, low flammability, high ionic density and low melting point. Ionic liquids have been known for more than a hundred years now [1], but research activities have taken off primarily since the late 1990’s and have since then continuously intensified. Ionic liquids find use in many energy-relevant devices, such as Li-ion batteries, capacitors and fuel cells [2,3]. Concerning the particular use in proton-exchange membrane fuel cells (PEMFCs), protic ionic liquids have been proposed as alternative materials for proton transport [4,5], based on the fact that protic ionic liquids may be able to shuttle protons and replace water to realize new electrolyte concepts. In practice, protic ionic liquids are considered to be competitive proton-conducting materials at temperatures above 80 °C, where water-based electrolytes cannot perform due to dehydration. Still today, hydrated perfluorosulfonic acid membranes (PFSA, i.e., membranes based on a perfluorinated polymer backbone to which side chains terminating with a sulfonic acid group are attached) are the state-of-the-art materials in commercial PEM fuel cells, and they all suffer from a limited temperature range of operation. Despite the huge interest that protic ionic liquids have attracted due to their anhydrous proton conductivity, their potential use in intermediate-temperature PEM fuel cells has also been challenged [6,7]. On a positive side, these works point to alternative strategies, such as the use of mixtures and systems where ionic liquids can support proton conduction through other roles.

Proton transfer is one mechanism of charge transport specifically investigated in protic ionic liquids while in the broader context that also includes aprotic ionic liquids, charge transport has been studied by focusing on the dynamics of the molecular species, i.e., the anions and cations. Standard methods to analyze molecular dynamics and charge transport in ionic liquids include nuclear magnetic resonance (NMR) spectroscopy and impedance spectroscopy, by which self-diffusion coefficients and ionic conductivity can be estimated, respectively. Molecular dynamics (MD) simulations can be performed to gain complementary insights on this matter [8]. Studies performed so far indicate that the molecular structure, intermolecular interactions, composition and confinement can all affect the macroscopically observed transport properties [9,10,11,12,13]. Moreover, the current understanding is that the vehicular mechanism of charge transfer dominates in ionic liquids, even in protic ionic liquids that may, in principle and under certain circumstances, also support other means of proton transfer such as hopping or the Grotthuss mechanism [14]. Our own works have shown that neither varying the alkyl chain length anchored to the imidazolium cation [9] nor mixing with water [15] results in charge-transport mechanisms significantly different from the vehicular one, whilst the addition of a base to a protic ionic liquid [16,17] or protic ionic liquids at nonstoichiometric composition [18] may enhance proton dynamics. In ionic liquids in general, there is a strong correlation between ionic conductivity and viscosity, which both depend on temperature in a way that is typically well described by the empirical Vogel–Fulcher–Tammann (VFT) relation. Previous comparative, systematic studies on the transport properties of ionic liquids have focused mainly on imidazolium-, ammonium- and pyridinium-derived cations, while less is known on ionic liquids based on triazolium, which are now addressed in this work.

Protic ionic liquids are a subclass of ionic liquids and can be obtained by proton transfer from a Brønsted acid to a Brønsted base, via a neutralization reaction [19]. This reaction is often presented as a straightforward and simple procedure, although in reality serious issues exist related to limiting the number of impurities, the water content and ensuring that the desired 1:1 ratio between acid and base is achieved. The presence of impurities or the realization of nonstoichiometric compositions will ultimately affect the physicochemical properties measured, shifting from the case of a truly pure liquid. Moreover, determining what these impurities really consist of, especially when present at very low content, is not always an easy experimental task. We have recently discussed these issues in depth and proposed a solvent-free synthesis protocol that resulted in highly pure and dry protic ionic liquids [20].

In that work, we focused on the synthesis of triazolium-based protic ionic liquids, benchmarking them against the properties of the better-known class of imidazolium-based ionic liquids. Triazole is an interesting analog to imidazole, due to the substitution of a C-H group for a N atom that results in a higher acidity, thereby we wanted to explore how this small change in the molecular structure of the cation would affect the properties of the resulting ionic liquids. In that previous work [20], along with reporting a new solvent-free synthesis procedure, we mainly investigated the properties of these compounds by experimental and computational methods. We found a strong effect of the cation, with the triazolium resulting in a more pronounced acidity than the imidazolium, whilst the triflate anion [TfO] resulted in a higher acidity when compared to the bistriflimide anion [TFSI].

In the study presented here, we aimed to investigate how these local structural differences possibly influenced the macroscopically measured transport properties, more specifically, self-diffusion and ionic conductivity. Moreover, with the aim of being rigorous in reporting the properties of ionic liquids, we emphasize the development of a specific setup that enables the measurement of the ionic conductivity as a function of the temperature inside a glovebox. This setup ensures that the investigated ionic liquids are kept free from moisture and any other type of contamination during the measurement; it also delivers highly reproducible values with very small errors. To our knowledge, this specific setup has not been reported before and will hopefully be reproduced in other laboratories for the purpose of characterizing small volumes of ionic liquids and other materials in their purest state. We also stress that triazolium-based protic ionic liquids are relatively new in the field, and in-depth studies of their transport properties are limited in number [21,22,23]. We believe that the comparative study reported here is a relevant contribution to the broader community interested in ionic liquids, regardless of the intended application. As a note on this line, triazolium-based ionic liquids have already attracted interest as cellulose solvents [24] and as charge carriers in all-organic batteries [22].

## 2. Materials and Methods

### 2.1. Materials

**Figure 1:**
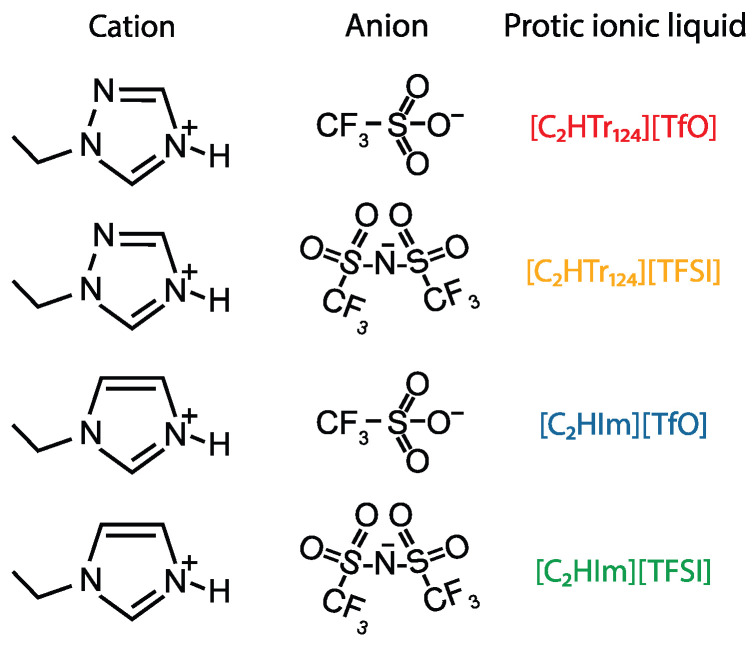
Molecular structure of the cations and anions constituting the four investigated protic ionic liquids. The color code used in this figure is the same as the one used in our related, precedent article [20] and is maintained throughout this paper. Abbreviations are as follows: [C${}_{2}$HTr${}_{124}$] is ethyltriazolium, [C${}_{2}$HIm] is ethylimidazolium, [TfO] is triflate and [TFSI] is bistriflimide.

The protic ionic liquids were prepared as described in great detail in reference [20]. We recall that a solvent-free synthesis procedure was used, which resulted in dry and pure protic ionic liquids (the water content was in all cases below 553 ppm and the purity higher than 98%). For clarity, the molecular structure of the constituting ions in the four protic ionic liquids is reproduced once again in Figure 1. Single-use conductivity standard sachets (12.88 mS/cm) were purchased from Mettler-Toledo.

### 2.2. Pulsed-Field Gradient NMR Spectroscopy

NMR studies were carried out using an NMR spectrometer (AVANCE III HD, Bruker, Billerica, MA, USA), operating at 14.1 T, equipped with a diff30 probe. The translational diffusivity was investigated at 307.0 K and the temperature was calibrated using an NMR tube with pure ethylene glycol prior to the experiments. Self-diffusion coefficients for both the anion and the cation of the four protic ionic liquids were obtained by using ${}^{1}$H and ${}^{19}$F 5 mm RF coil insets, respectively. The specific parameters were individually set for each nucleus. For the ${}^{1}$H experiments, a 17.0 μs 90° pulse, 0.41 s acquisition time, 12 s recycle delay, and 40 kHz spectral width were used. For the ${}^{19}$F experiments, a 13.5 μs ${}^{19}$F 90° pulse, 0.83 s acquisition time, 2.5–5 s recycle delay, and 20 kHz spectral width were used. The chemical shifts were calibrated using tetramethylsilane as an external reference.

For the diffusion experiments of the pulsed-field gradient (PFG) type, a double stimulated echo (diffDste) was used in order to suppress possible convection effects in the ionic liquids at elevated temperatures. The measurements were conducted using 16 gradient steps. The parameters for both ${}^{1}$H and ${}^{19}$F were optimized to obtain a full signal attenuation, including Δ = 100 ms (${}^{1}$H), Δ = 50 ms (${}^{19}$F), δ = 2 ms and linear increments of *g* to *g*${}_{max}$ of 400 G/cm (${}^{1}$H) and 600 G/cm (${}^{19}$F). The correct calibration of the gradient amplifier was verified by obtaining the self-diffusion coefficient of HDO traces in a standard sample of pure D${}_{2}$O [25]. The self-diffusion coefficients of the individual ions were calculated by fitting the Stejskal–Tanner equation [26] to the signal’s integral intensity attenuation data according to

(1) $$I=I_{0}\times e^{-D{\left(\gamma g\delta \right)}^{2}\left(\Delta -\delta /3\right)}$$

where *I* is the signal intensity, *I*${}_{0}$ the signal intensity at zero gradient, Δ the diffusion time, δ is the gradient pulse duration, γ is the gyromagnetic ratio of the nucleus studied, and *g* is the gradient strength. A least-squares fitting, using *k* = (γgδ)${}^{2}$(Δ−δ/3), of parameters *I*${}_{0}$ and *D* was applied with a nonlinear dependence using the Levenberg–Marquardt method [27]. The presented errors (≤4 %) show the standard deviation (95 % confidence interval) of the fitted diffusion coefficient.

**Figure 2:**
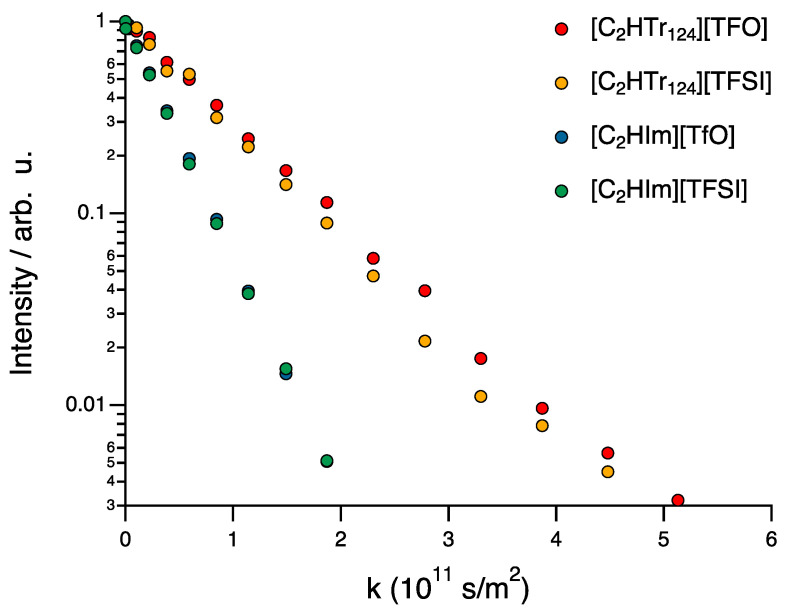
Intensity decay as a function of k, shown in a log scale, for the NMR resonance of the hydrogen atom of the ethyl chain anchored to the cation, i.e., ethyltriazolium or ethylimidazolium.

The intensity decay of the NMR signal as a function of *k* is shown in Figure 2 for the representative case of the methyl group of the cation, in all four protic ionic liquids. The decays display a strictly monoexponential pattern, indicating a single population of diffusing molecular species. The monoexponential slope is also observed for all other hydrogen and fluorine atoms in all samples.

### 2.3. Vibrational Spectroscopy

Raman spectra were collected at room temperature using a Raman spectrometer (InVia Reflex, Renishaw, Wotton-under-Edge-Gloucestershire, UK). A 785 nm diode laser was used as the excitation source (using 10% of nominal laser power, hence ∼30 mW of power, measured along the optical path before the objective/sample stage). A diffraction grating with 1200 lines/mm was selected. A Leica 50× objective with a numerical aperture (NA) of 0.75 was used to focus the laser onto the sample. Eight scans of 10 s each were acquired covering the spectral range 100–4000 cm${}^{-1}$. The spectrometer was calibrated prior to the measurements using a Si wafer and calibrating its first-order vibrational mode to be at 520.6 cm${}^{-1}$. The ionic liquid samples were prepared inside a glovebox and placed in a custom-made air-tight sample holder, designed to minimize exposure to moisture before and during the collection of Raman spectra (see Figure S1 in the supporting information of reference [20]). All spectra were baseline-corrected and normalized to the most intense signature for comparison and further analysis.

### 2.4. Ionic Conductivity

**Figure 3:**
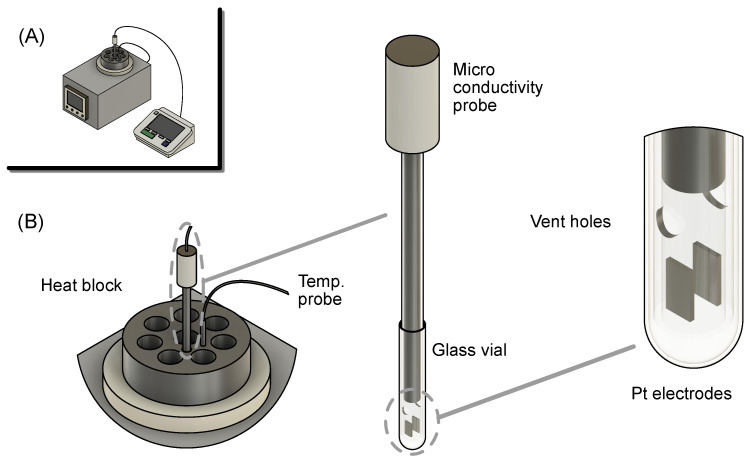
Ionic conductivity setup. (**A**) Entire setup inside the glovebox. Polar Bear Plus to the left, conductivity meter to the right. (**B**) Zoomed-in details of the components of the setup.

The ionic conductivity of the protic ionic liquids was measured inside a N${}_{2}$-filled glovebox (MBRAUN UNIlab Plus Eco glovebox with an MB-LMF II solvent absorber system) using a special setup, designed to enable the measurement of conductivity values of small volumes (<0.5 mL) of ionic liquids, without the interference of atmospheric moisture; refer to Figure 3 for an illustration of the setup. An extended discussion about the development and use of this setup can be found in the Results and Discussion section. In practice, a microconductivity probe (Mettler-Toledo InLab 751 - 4 mm with a SevenCompact S230 conductivity meter; cell constant: 1.0 cm${}^{-1}$) was inserted into a small glass tube vial (35 mm tube with a 5 mm inner diameter) containing the ionic liquid sample. The glass vial was in turn placed into a hole made in an aluminum heating block, which was kept at the desired temperature using a magnetic stir plate with heating and cooling capabilities (Cambridge Reactor Design, Polar Bear Plus device), especially suited to be used inside a glovebox. The temperature of the sample was measured using an external temperature probe (Polar Bear Plus external temperature probe) placed in a hole nearby.

The ionic conductivity setup was calibrated using single-use sachets of an aqueous conductivity standard of 12.88 mS/cm from Mettler-Toledo. The instrument was calibrated in open air at a sample temperature of 25 °C. The calibration standards were placed in the same small glass tubes used for measuring the conductivity of the ionic liquids, using a similar temperature control setup to the one used inside the glovebox. Still outside the glovebox, triplicate samples of the conductivity standard were measured, to ensure successful calibration. The conductivity was measured to be 12.89 ± 0.02 mS/cm.

Inside the glovebox, triplicate samples of a commercial ionic liquid, [C_2_HIm][TFSI] (from IOLITEC), had their ionic conductivities measured at 25 °C, in order to estimate the variation in measured values for an ionic liquid sample. The ionic conductivity was measured to be 4.633 ± 0.002 mS/cm. The conductivities of all ionic liquids were measured using single samples (due to the limited volume of sample material) at temperatures ranging from 25 to 80 °C.

### 2.5. Computational Results

In order to determine hydrogen-bonding energies ($E_{\mathrm{HB}}$), the quantum theory of atoms in molecules (QTAIM) analysis [28] was performed using Multiwfn 3.7 software [29]. DFT data (ωB97X-D3BJ/cc-pVTZ/CPCM-Etanol level of theory) from the calculations performed for our previous work [20] were used as the input. These energies were calculated using the definition presented by Espinosa et al. [30], which correlates potential electron energy density at the bond critical point, i.e., $V(r_{\mathrm{BCP}})$, with $E_{\mathrm{HB}}$ (Equation (2)). The molecular geometry data (bond lengths) presented here are the same as those presented in the previously referenced work [20].

(2) $$E_{\mathrm{HB}}=\frac{1}{2}V\left(r_{\mathrm{BCP}}\right)$$

## 3. Results and Discussion

Before discussing the data acquired using the ionic conductivity setup described above, it is important to describe our motivation for developing it, how to properly use it and the limitations it may have. As a result of our previous work on the synthesis of new protic ionic liquids [20], we finalized the project with very limited volumes of the synthesized ionic liquids, implying that any additional analysis to be performed on those materials would need to be conducted on very small volumes. This situation is common for other researchers working on the development of new ionic liquids, where only small amounts are synthesized. Another important aspect of conductivity measurements is their sensitivity to adsorbed moisture, which means that a sample analyzed in open air will constantly change its conductivity due to water uptake. This is the case for most ionic liquids, which tend to be very hygroscopic, and protic ionic liquids in particular. This issue can be resolved by running the measurements inside a glovebox; however, this introduces the problem of temperature control. This is why we chose to use the Polar Bear Plus equipment, which is perfectly suited to control the temperature of our samples inside a glovebox. The equipment allows the cooling and heating of the sample and, consequently, the analysis of the sample at various temperatures. This setup easily allows for the measurement of conductivities between 0 to 100 °C, a limitation imposed by the conductivity probe (according to the manufacturer). This could possibly be extended from −40 to 150 °C (the range of operation of the Polar Bear Plus instrument), but no tests have yet been conducted outside the range of temperatures reported in this publication. In terms of the practical use of the setup, there are a few factors that must be considered in order to acquire reliable data. Despite the triplicate data (previously mentioned in the Conductivity Measurements section) presenting only minor variations between data points (standard deviation of 0.002 to 0.02 mS/cm), at the early stages of development, this setup revealed significant errors. This was due to small bubbles or debris trapped on the Pt electrodes. These issues are usually not present with other setups, since most times, the volume of sample (and the electrode area) is much larger and not static (typically under agitation). Thankfully, these problems could be easily solved by rigorously cleaning the electrodes and being careful to fully wet the electrodes with the liquid sample. We recommend filling the sample tube with sufficient liquid, such that it is above the vent holes’ level (Figure 3). In order to avoid bubbles, the probe must be slowly inserted into the tube, so that the air can escape through the vent holes of the probe. The absence of bubbles can be verified visually, before inserting the probe and vial in the heat block. For ionic liquids of high viscosity, it might be useful to preheat the sample before inserting the probe inside the tube. A final recommendation is simply running the experiment in triplicate, since an outlier value could then be easily spotted. Unfortunately, this could not be done in this work due, again, to the limited volume of sample available.

**Figure 4:**
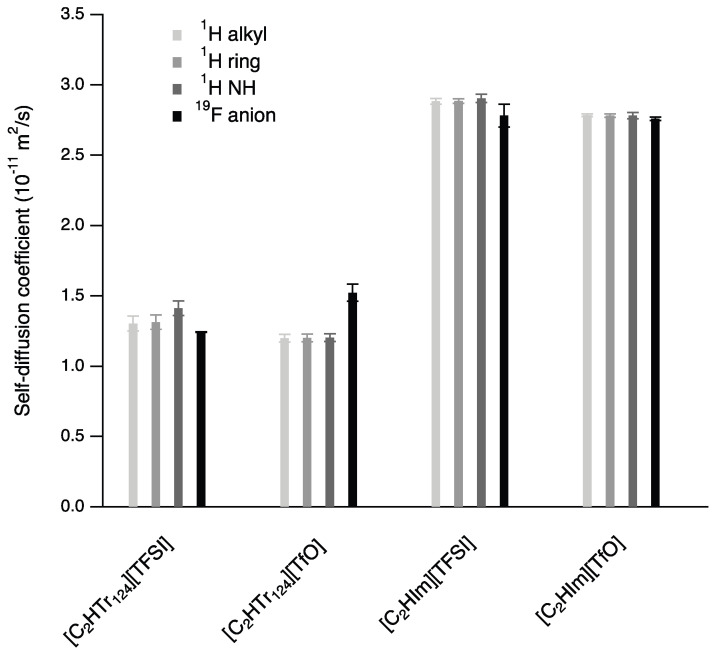
Self-diffusion coefficients estimated from PFG NMR measurements for different ${}^{1}$H and ${}^{19}$F resonances and for all four protic ionic liquids at 34 °C. The errors show the standard deviation of the fitted diffusion coefficient.

Another technique commonly used to probe the transport properties of these compounds is PFG NMR, which can be used to measure self-diffusion with chemical selectivity. As mentioned in Section 2.2, the intensity decay, as a function of *k*, of the investigated NMR resonances was monoexponential for all four protic ionic liquids, indicating that there was one single population of diffusing species and that on average, the representative self-diffusion coefficient could be extracted from each resonance, i.e., for specific hydrogen atoms. The self-diffusion coefficients estimated from ${}^{1}$H PFG NMR measurements are summarized in Figure 4, distinguishing values obtained for the exchangeable hydrogen in the NH bond, the hydrogen atoms in the ring structure and the hydrogen atoms in the ethyl chain. In addition, the self-diffusion coefficient of the associated anion, [TfO] or [TFSI], is also reported, which was retrieved from ${}^{19}$F PFG NMR measurements.

For all four protic ionic liquids, the reported values were very similar for the three types of hydrogen atoms, indicating that the exchangeable proton did not diffuse significantly differently from the parent molecule. Contrarily, this similarity revealed that the exchangeable proton diffused primarily with the cation, in a so-called vehicular mechanism that in turn was governed by the viscosity of the fluid in which the ions moved. The absence of exceptional—i.e., different from purely vehicular—mechanisms of proton transfer has been reported before for a number of pure protic ionic liquids based on alkyl-imidazolium cations (with the alkyl chain varying from ethyl to dodecyl) [9] as well as for a water–protic ionic liquid mixture [15]. Despite this insight, we stress the importance of further investigating the proton dynamics in related systems, such as base or acid-doped protic ionic liquids or ionic systems derived from nonstoichiometric acid:base ratios [6,16,17,18]. Figure 4 also shows that the self-diffusion coefficients of cations and anions in one specific protic ionic liquid were very close to each other; only for the protic ionic liquid [C${}_{2}$HTr${}_{124}$][TfO] was the self-diffusion coefficient of the anion slightly higher than that of the cation.

**Figure 5:**
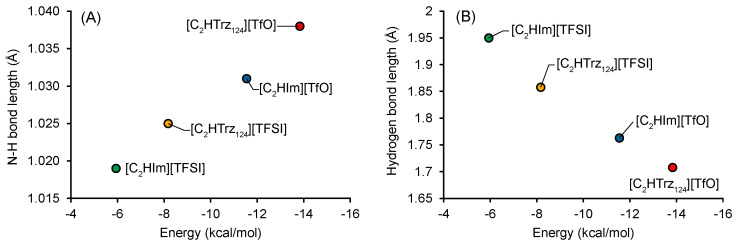
N–H bond length (**A**) and hydrogen bond length (**B**) as a function of hydrogen-bonding energy ($E_{\mathrm{HB}}$). Calculated at the ωB97X-D3BJ/cc-pVTZ/CPCM-Ethanol level of theory.

An important observation was the strong effect of the cation on diffusivity. The [C${}_{2}$HTr${}_{124}$] cation had a self-diffusion coefficient almost half of that estimated for the [C${}_{2}$HIm] cation instead. In comparison, the effect of changing the [TfO] anion for [TFSI] was much smaller. This clear difference can be rationalized by the differences in molecular descriptors for these four ionic liquids. As previously reported [20], triazolium protic ionic liquids have a higher acidity compared to imidazolium, which is evident by their longer N-H bonds. This elongation is also correlated with a shortening of the hydrogen bond between the ions, indicating stronger hydrogen bonds (Figure 5). We speculate that this increased hydrogen bonding can be one of the factors resulting in the lower diffusivity and ionic conductivity (see below) of the triazolium-based ionic liquids.

**Figure 6:**
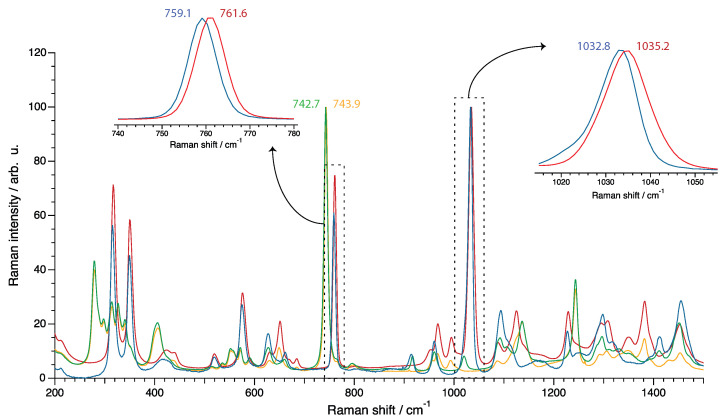
Raman spectra recorded at room temperature for the four protic ionic liquids, i.e., [C${}_{2}$HIm][TFSI] (green), [C${}_{2}$HTr${}_{124}$][TFSI] (yellow), [C${}_{2}$HIm][TfO] (blue) and [C${}_{2}$HTr${}_{124}$][TfO] (red). The insets (normalized) show zoomed-in regions to visualize relative frequency shifts of [TfO]-related vibrations.

Further insight into the nature of intermolecular interactions can appropriately be gained by vibrational spectroscopy, preferably by Raman spectroscopy since it can provide a higher spectral resolution than infrared spectroscopy. The Raman spectra recorded for all four protic ionic liquids are shown in Figure 6, showing the spectral range 200–1500 cm${}^{-1}$. The strongest vibrations observed were those arising from the anions, more specifically the mode at ∼742 cm${}^{-1}$ (ν${}_{s}$ S-N-S and ν${}_{s}$ CF${}_{3}$ in [TFSI]), the mode at ∼760 cm${}^{-1}$ (ν${}_{s}$ CF${}_{3}$ in [TfO]) and the mode at ∼1035 cm${}^{-1}$ (ν${}_{s}$ SO${}_{3}$ in [TfO]) [20]. The position of these vibrational modes and their sensitivity to the coordination of the anion was investigated in pioneering works based on both experiments and theoretical calculations [31,32,33]. Following studies further demonstrated the appearance of an additional, blue-shifted vibrational mode when adding alkali [TFSI] salts to an imidazolium-based ionic liquid [34]. This new mode was associated with the formation of alkali–[TFSI] complexes. Interestingly, the extent of the shift between the lower frequency peak assigned to “free” [TFSI] (so-called “free” due to the relatively weak ionic coordination) and this new one was related to the size (and therefore the interaction strength) of the alkali metal. The biggest shift was found for Li${}^{+}$ and the smallest for Cs${}^{+}$, which blue-shifted by only ∼1 cm${}^{-1}$ from the “free” [TFSI] anion. This shows that weakly coordinating cations are expected to have a minor but measurable effect on the shift of these vibrational modes.

The Raman spectra shown in Figure 6 revealed single component modes, centered at ∼1033 cm${}^{-1}$ for [C${}_{2}$HIm][TfO] and at ∼1035 cm${}^{-1}$ for [C${}_{2}$HTr${}_{124}$][TfO] (see right inset). On these grounds, the small but measurable blue shift for the [TfO] anion of the triazolium-based compound may imply a stronger cation–anion interaction when compared to its imidazolium counterpart; the same conclusion could be drawn from the frequencies of the mode at around 760 cm${}^{-1}$ (shown in the left inset). The frequency analysis of the vibration of [TFSI] at ∼742 cm${}^{-1}$ indicated the same trend for the strength of cation–anion interactions (743.9 cm${}^{-1}$ in [C${}_{2}$HTr${}_{124}$][TFSI] vs. 742.7 cm${}^{-1}$ in [C${}_{2}$HIm][TFSI]).

In the literature, these frequencies (i.e., 740–744 cm${}^{-1}$ for [TFSI] and 756–759 cm${}^{-1}$ for [TfO]) have been used many times to extract information about the nature of anion–cation interactions [31,32,34,35,36,37,38]. These trends, reported for systems based on, for instance, polymers, ionic liquids and classical organic electrolytes, may be extended, with caution, to also analyze the difference in the strength of anion–cation interactions established by [TFSI] and [TfO] in protic ionic liquids.

**Figure 7:**
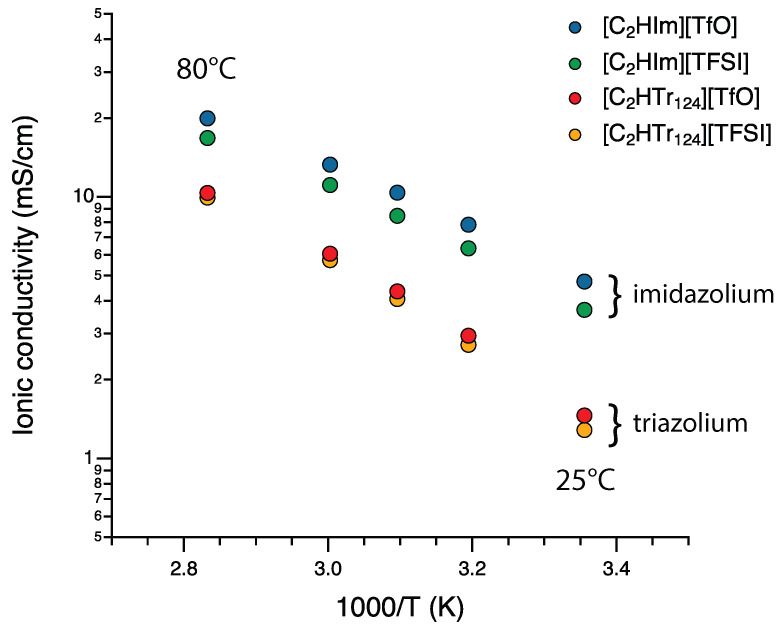
Ionic conductivity values (shown in a log scale), measured for the four protic ionic liquids in the dry and inert atmosphere of a glovebox, as a function of temperature.

With the hypothesized correlation between a slower molecular diffusion and stronger cation–anion interactions, it becomes interesting to analyze whether the found differences are also reflected in the ionic conductivity. The ionic conductivity in these ionic liquids was measured at variable temperatures, in the inert atmosphere of a glovebox to avoid overestimation due to moisture. More precisely, the ionic conductivity was measured from 25 up to 80 °C, as shown in Figure 7 in the representation of an Arrhenius plot. It is observed that the ionic conductivity also showed a stark dependence on the type of cation, with the protic ionic liquids based on [C${}_{2}$HTr${}_{124}$] revealing a lower ionic conductivity than those based on [C${}_{2}$HIm], in accordance with the results obtained by PFG NMR. In addition, the conductivity data showed a nonlinear trend in the Arrhenius plot, which is typical for the broader family of ionic liquids and reflects a charge-transfer mechanism primarily governed by viscosity. The effect of the anion was less pronounced but tended to result in slightly higher ionic conductivity when substituting [TFSI] with [TfO]. In the case of the ionic liquids based on [C${}_{2}$HIm], the positive effect of [TfO] was measurably larger (see also values given in Table 1).

The temperature dependence of the ionic conductivity was further analyzed by fitting the experimental data with the empirical equation

(3) $$\sigma =\sigma_{0}\cdot e^{-(\frac{D\cdot T0}{T-T0})}$$

The expression in Equation (3) is also known as the VFT (Vogel–Fulcher–Tammann) equation, in which σ${}_{0}$ is the ionic conductivity extrapolated for infinite temperatures, *D* is a parameter related to fragility (the strength parameter), *T*${}_{0}$ is the Vogel temperature, while *T* is the absolute temperature expressed in Kelvin. Into this fitting procedure, we included an additional data point by setting the ionic conductivity to the assumed value of 10${}^{-15}$ S/cm at the temperature T${}_{g}$. This data point was not accessible with the experimental setup used inside the glovebox. This assumption holds true for systems in which the ionic conductivity (σ) and the structural relaxation time (τ) are highly coupled and inversely proportional. Due to this inverse proportionality, the variation by 16 orders of magnitude for the relaxation time τ (notably between 10${}^{-14}$ s and 100 s) should translate into an equally large gap between the value of σ estimated for infinite temperatures (∼1 S/cm) and the value of σ associated with the glass transition temperature (hence 10${}^{-15}$ S/cm). This coupling was addressed in reference [39], which mentions the fact that at T${}_{g}$, the structural relaxation time reaches a fixed value of about 100 s. Hence, based on the Stokes–Einstein and Nernst–Einstein relations, the ionic conductivity would be about 10${}^{-15}$ S/cm at T${}_{g}$ (“and would be immeasurable by normal methods”). The value of ∼1 S/cm is typically associated with the temperature of “normal” viscosity [40] and has also been found from the extrapolation to infinite temperatures of experimental data of the conductivity of many ionic liquids reported in the literature (i.e., the pre-exponential factor $\sigma_{0}$ in Equation (3)). Interestingly, for a series of imidazolium-based ionic liquids, a very good match was found between the temperatures at which τ is equal to 100 s and σ is equal to 10${}^{-15}$ S/cm [41]. Although the full coupling described above cannot be assumed *a priori* for any ionic liquid system (molten salts included), with the lack of experimental data in a wider temperature interval than the one available to us, we made this reasonable assumption for the ionic liquids investigated here as well. The results presented below are thus a consequence of setting σ(T${}_{g}$) equal to 10${}^{-15}$ S/cm, which provides the opportunity to extend the discussion of ionic conductivity to the concept of fragility.

The T${}_{g}$ values used in this approach were those previously measured for these protic ionic liquids by DSC using a fast cooling rate of 10 K/s [20]; these are also reported in Table 1 together with representative conductivity and diffusivity values. We note that the protic ionic liquid [C${}_{2}$HIm][TfO] easily crystallized upon cooling (during both fast and slow cooling scans), whereas a T${}_{g}$ value was not reported nor available for fitting data in the lower-temperature range. A very important aspect of this approach is that by including the conductivity value at T${}_{g}$, hence extending the VFT fitting to very low temperatures, the fragility of the protic ionic liquids can be accessed [41,42]. Moreover, whilst for conductivity values measured within a limited temperature range, a good fit could be achieved with a number of *D* and T${}_{0}$ combinations, when including the additional point at T${}_{g}$, the accuracy, and hence the usefulness, of the fitting was significantly enhanced.

Fragility is a property of glass-forming materials that characterizes how rapidly the dynamics slow down upon cooling towards the glass transition temperature T${}_{g}$ or, vice versa, how rapidly they change upon heating from T${}_{g}$. The degree of deviation from the Arrhenius behavior as a classification tool between strong and fragile materials was originally proposed by A. Angell, typically using a so-called Angell plot (i.e., a T${}_{g}$-scaled Arrhenius plot) [43]. In an Angell plot, the kinetic fragility index *m* can be estimated from the slope of viscosity (or relaxation time) close to T${}_{g}$. Another fragility parameter that can be used to distinguish between strong and fragile materials is *D*, which can be obtained by fitting the dependence of the viscosity (or relaxation time) on the inverse temperature using the empirical VFT formula. More recently, the change of ionic conductivity with temperature has also been proposed as a reasonable and viable tool for investigating the fragility of ionic liquids, under the assumption that the ionic motion remains coupled to viscosity [41] (In this context, one should note that flash differential scanning calorimetry can also be used to estimate the kinetic fragility of ionic liquids/materials [44]). To summarize, an Arrhenius plot of either viscosity, conductivity, or relaxation time can be used to distinguish materials that are fragile or strong, fragility being represented by strong curvatures and strength appearing as a close-to-linear behavior. Accordingly, low values of *D* in the VFT expression reflect fragile materials, while large values of *D* are representative of stronger materials (see also the illustrative figures in reference [41]) (One should be aware of the fact that the *D* value obtained from fitting viscosity (or relaxation time) data can be different from that obtained from fitting conductivity data, due to the slightly different activation energy associated to these two properties). As a final note, the fragility parameters *m* and *D* are related through *m* = 16 + 590/*D* [45]. In particular, the fragility of ionic liquids has been shown to be a fundamental parameter to understand, and if possible to tune, since it eventually determines the ionic conductivity achievable at room temperature or above, i.e., the temperatures at which real electrochemical devices operate [41].

**Figure 8:**
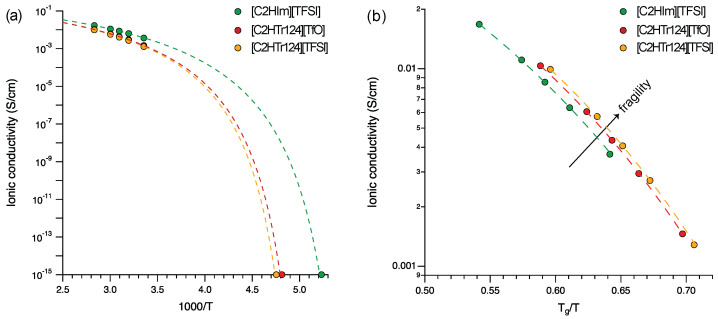
(**a**) Arrhenius plot of the ionic conductivity displayed by the three glass-forming protic ionic liquids. (**b**) T${}_{g}$-scaled plot (Angell plot) with the same experimental data shown in (**a**). Dashed lines are fits to the experimental data.

In the Arrhenius plot of Figure 8a, the conductivity values measured for the three protic ionic liquids that form a glass upon cooling are presented, including the conductivity values at T${}_{g}$ (set to 10${}^{-15}$ S/cm). Dashed lines are the best fits to the experimental data. Again, the values of the ionic liquids based on the [C${}_{2}$HTr${}_{124}$] cation tended to merge and showed a trend distinguished from that of the ionic liquid based on [C${}_{2}$HIm]. The fitting parameters reported in Table 2 reveal that the *D* values were not significantly different for the three ionic liquids, varying between ca. 3.3 and 3.5. The corresponding fragility parameter *m* varied from 198 to 186. Other works available in the literature treating protic ionic liquids based on, e.g., alkylammonium [46], pyrrolidinium [47], guanidinium [48] or alkylimidazolium [49] have reported fragility values from the analysis of ionic conductivity that would correspond to *D* values similar to the ones that we report here. Within our series, [C${}_{2}$HIm][TFSI] had a slightly higher value and should therefore be classified as a less fragile liquid than [C${}_{2}$HTr${}_{124}$][TFSI] or [C${}_{2}$HTr${}_{124}$][TfO].

As mentioned above, a T${}_{g}$-scaled plot is an alternative way to verify whether this is the case, since materials are then compared by normalizing the temperature scale to the glass transition temperature, which is significantly different for the triazolium- and imidazolium-based ionic liquids investigated here. Such a plot is given in Figure 8b and confirms that the ionic liquid [C${}_{2}$HIm][TFSI] was slightly stronger than the other two triazolium-based protic ionic liquids. This difference was very small but reliable, given the minute errors in the measured conductivity values. Moreover, this higher fragility is important and explains why in a T${}_{g}$-scaled plot, the triazolium-based ionic liquids display a slightly higher ionic conductivity despite their higher glass transition temperature (−62 and −65 °C vs. −82 °C). In other words, our data analysis demonstrates, in line with the discussion raised earlier by Sippel et al. [41], that a low T${}_{g}$ and a high fragility are both important for determining the ionic conductivity achievable at higher temperatures. This strengthens the knowledge that a favorable combination of *D* and T${}_{g}$ values is desirable for use in real applications. Finally, considering a larger span of values where *D* may, for example, vary from 4 to above 40 [41], we can conclude that the three protic ionic liquids investigated in this work all belong to the family of extremely fragile liquids. We finally aim to remark that we do not have enough information to speculate on the origin of the fragility differences in these protic ionic liquids, especially since the question of what exactly determines fragility in glass-forming materials has for years remained a puzzling question [50]. One aspect that has been analyzed though, for protic ionic liquids based on the decahydroisoquinolinium cation, is the correlation between fragility and ionicity [42], finding that fragility decreases with an increase in ΔpK${}_{a}$. This would make sense with the trend found here, with the triazolium protic ionic liquids having a smaller ΔpK${}_{a}$ and a concomitant higher fragility, compared to imidazolium.

## 4. Conclusions

This work was focused on the analysis of the transport properties of a series of protic ionic liquids synthesized by our group, previously investigated mainly from the viewpoint of local structural properties. Here, the self-diffusion of molecular species, ionic conductivity, fragility and intermolecular interactions were investigated instead. The ionic conductivities were measured with high accuracy using a reliable new setup, which enabled the measurement of the ionic conductivity of small volumes of ionic liquids under an inert atmosphere and at variable temperatures.

We found that the cation’s structure had a stark effect on the mobility of the ions, with the protic ionic liquids based on triazolium showing a lower diffusivity than those based on imidazolium. We hypothesized that one reason for that difference in transport properties was the stronger hydrogen bonding, which was in agreement with the findings of our previous study [20]. Nevertheless, no exceptional proton-transfer mechanisms—i.e., significantly different from the vehicular mechanism—were detected by PFG NMR, for any of the protic ionic liquids investigated. The analysis of Raman spectra corroborated our conclusion that stronger interactions were established between the anions and the triazolium cation, compared to the case of imidazolium.

The temperature dependence of the ionic conductivity analyzed in conventional and T${}_{g}$-scaled Arrhenius plots also revealed a clear effect of the cation, with the triazolium-based ionic liquids having similar T${}_{g}$ and fragility values. Moreover, the two triazolium-based protic ionic liquids had a slightly higher fragility than the one based on imidazolium. This differentiation was made possible by including into the VFT fit a conductivity value associated with the glass transition temperature T${}_{g}$, which should be seen as an interesting approach in studying ionic liquid systems. We discussed how a higher fragility may be beneficial for real applications and to compensate for the otherwise slightly higher, hence less favorable, T${}_{g}$ value. Finally, our results indicated that the choice of the anion, that is [TFSI] or [TfO], had a lesser effect on the dynamics in these protic ionic liquids.

## Figures and Tables

**Table 1:** Selected properties of the protic ionic liquids investigated, i.e., glass transition temperature (T${}_{g}$), ionic conductivity (σ) and self-diffusion coefficient (D${}_{cat}$). The self-diffusion coefficient is that of the cation, D${}_{cat}$, the value reported here being the one estimated from one of the hydrogen atoms of the heterocyclic ring. ${}^{a}$ Values previously reported in reference [20]. * [C${}_{2}$HIm][TfO] is metastable at 25 °C due to its melting point being close to that temperature [20].

Property	T${}_{g}$	σ	D${}_{\mathrm{cat}}$
Units	(K)	(mS/cm)	(10${}^{-11}$ m${}^{2}$/s)
Note	${}^{a}$	@ 25 °C	@ 34 °C
[C${}_{2}$HIm][TFSI]	191	3.693	2.885
[C${}_{2}$HIm][TfO]	n.a.	4.745 *	2.781
[C${}_{2}$HTr${}_{124}$][TFSI]	211	1.284	1.313
[C${}_{2}$HTr${}_{124}$][TfO]	208	1.458	1.200

**Table 2:** Fitting parameters obtained by fitting the experimental conductivity data with the empirical VFT relation given in Equation (3). Fitting parameters are reported as values ± one standard deviation. The fragility parameter *m* has also been calculated (using m=16+590/D [45]).

Ionic Liquid	D	T${}_{0}$ (K)	$\sigma_{0}$ (S/cm)	m
[C${}_{2}$HIm][TFSI]	3.4741 ± 0.0232	174.0	0.4926 ± 0.0122	186
[C${}_{2}$HIm][TfO]	-	-	-	-
[C${}_{2}$HTr${}_{124}$][TFSI]	3.2471 ± 0.0106	192.5	0.4882 ± 0.0067	198
[C${}_{2}$HTr${}_{124}$][TfO]	3.3027 ± 0.0074	190.0	0.4865 ± 0.0046	195

## Data Availability

The data presented in this study are available upon request from the corresponding author.
